# My home is where my health is: narratives on health promotion from older people living at home

**DOI:** 10.1080/17482631.2025.2518668

**Published:** 2025-06-15

**Authors:** Therese Hugøy, Helle K. Falkenberg, Grethe Eilertsen, Marit Skraastad, Anette Hansen

**Affiliations:** a Faculty of Health and Social Sciences, University of South-Eastern Norway, Porsgrunn, Norway; b USN Research Group of Older Peoples’ Health, University of South-Eastern Norway, Drammen, Norway; c National Centre for Optics, Vision and Eye Care, Faculty of Health and Social Sciences, University of South-Eastern Norway, Kongsberg, Norway; d Faculty of Health and Social Sciences, University of South-Eastern Norway, Drammen, Norway; e Telemark, The Centre for Development of Institutional and Home Care Services (USHT Telemark), Porsgrunn, Norway

**Keywords:** Older people, meaning of home, living at home, ageing in place, narrative, health promotion, home care nursing, home-dwelling, sense of coherence

## Abstract

**Purpose:**

Many older people want to live at home for as long as possible, and the aging population is highlighting the importance of understanding what they require for their lives to be good. This study explored how older recipients of home care experience the meaning of home from a health-promoting perspective.

**Methods:**

Narrative interviews were conducted with 10 people aged 78–103 years living at home and receiving home care nursing. We conducted a thematic narrative analysis, and based on the results chose a salutogenic perspective to shed light on the health-promoting perspective.

**Results:**

The primary theme identified was “My home is where my health is.” It reflects a compellation of the four subthemes emerging from the data: (1) my home promotes independence and autonomy, (2) my home promotes identity and self, (3) my home promotes being active, and (4) the support I receive is pivotal to promoting health and life at home. These themes are pivotal to health promotion and well-being in old age.

**Conclusions:**

Living at home contributes to meaningfulness, manageability, comprehensibility, and a sense of coherence. This promotes health and well-being for older people living at home if they receive both formal and informal support.

## Introduction

The home has been identified as a place where the day-to-day routines and practical tasks are performed, and has great personal and symbolic value (Thorsen, 2008). The home is our foundation in life, and where we naturally return to (D. G. Rowles & Chaudrey, 2005).

The concept of home can be difficult to define in research. It can be understood as a physical place, house, or town, a relationship, a feeling at home, and as an experience of being at home (Gillsjö & Schwartz-Barcott, 2011). The term “home” as a scientific concept can be defined as a place where an individual experiences emotional attachment, comfort, security, and a sense of homeliness. It is where one feels a sense of belonging and familiarity (Gillsjö & Schwartz-Barcott, 2011). Many people relocate to new homes multiple times throughout their lives, and experience to feel belonging and at home in different places and locations (Hatcher et al., 2019; Mahler et al., 2014; Munkejord et al., 2018). There is a paucity of research on the health promotive aspects of living in one’s own home during old age, particularly regarding recent studies on the topic. Additionally, there is a lack of research that includes perspectives from older people themselves.

Health authorities in Norway emphasize that the home is the best place to grow old (2019), which seems to be consistent with the views of many older people (Bjerkmo et al., 2021; Sabia, 2008; Tanner et al., 2008). As long as they feel safe, most older people want to live at home even when they become frail and need comprehensive professional care (Bjerkmo et al., 2021; Dale et al., 2001). Supporting older people to continue living at home for as long as possible has become a priority for policymakers in many countries, including Norway (2023).

There is considerable discussion about the term “health” in gerontological research. However, there is a general consensus that the concept of health cannot meaningfully be defined as the absence of disease for older persons due to the higher prevalence of diagnosable disorders in this population (Smith et al., 2002). Health can be described as a broad, complex, multidimensional phenomenon with physical, mental, and social dimensions. Normal age-related changes, chronic diseases, disabilities, and other types of limitation result in older adults having different perceptions and experiences of health than younger adults (Tan et al., 2014). A review of older people’s perceptions of their health found that they feel healthy when they can do things independently, do not have or can manage symptoms, can accept and adjust to changes with optimism, and have connectedness with others as well as sufficient energy (Song & Kong, 2015).

Health promotion represents a shift in public health from targeting individual risk factors or behaviours to addressing determinants of health and enabling communities to participate in improving their health on the own (Kickbusch, 2003; Mittelmark et al., 2008). In this study we used the WHO definition that states that “health promotion is the process of enabling people to increase control over, and to improve their health.” The home is a setting that influences the health status and affects the everyday lives of older people (WHO, 1986). WHO subsequently introduced the concept of healthy ageing, defined as “the process of developing and maintaining the functional ability that enables well-being in old age” (WHO, 2020).

Ageing in place refers to the ability of older adults to live in their own homes and communities for as long as possible, delaying entry into long-term care facilities (Marek et al., 2005; Rantz et al., 2011). This emphasizes the centrality of the private home for all older adults, including those with health challenges. Ageing in place has emerged as a central topic in gerontological research, driven by the significant demographic changes and the rapid growth of the older population. Importantly, ageing in place encompasses more than just a geographical location; it also encompasses social ties, psychological well-being, and the quality of relationships that older adults maintain (Pani-Harreman et al., 2021). However, research has also demonstrated that ageing in place can place significant strain on the informal social support networks of older adults (Horner & Boldy, 2008; Melilla et al., 2024) and lack of access to necessary services and support can reduce quality of life (Vanleerberghe et al., 2017). Physical barriers in the home, social isolation, and lack of informal support can undermine the ability to live independently. The home can become a place for loneliness and vulnerability (Munkejord et al., 2018; A. Sixsmith & Sixsmith, 2008). Brim et al. (2021) explored barriers older adults were facing when trying to age in place and the respondents reported that issues like stair safety, lighting, and general fall hazards were common concerns.

The meaning of home and the experience of living at home among older people have been explored from different perspectives (Dahlin-Ivanoff et al., 2007; Gillsjö et al., 2011; Ness et al., 2014; Swenson, 1998; Wiles et al., 2012). Within environmental psychology and gerontology, there seems to be a consensus that the meaning of home among older adults is related to aspects of physical, social, and personal bonding (D. G. Rowles & Chaudrey, 2005).

Kylén et al. (2019) found that the meaning of home in younger older people in Sweden becomes progressively more important after retirement. The idea of what constitutes a home for older people largely focuses on whether the living environment is accessible and practical (D. G. Rowles & Chaudrey, 2005). Some studies have investigated future housing needs for the elderly with the aim of tailoring future services (Bigonnesse et al., 2014; Bjerkmo et al., 2021). Other studies have explored barriers that older people experience living at home (Brim et al., 2021; Eilertsen et al., 2016; Falkenberg et al., 2019; Pettersson et al., 2020; Portacolone, 2013), focusing on safety and physical barriers in the home environment.

A literature review of studies into the home being a place for health promotion found that older adults could be empowered if they were supported in identifying and implementing their own health-promoting strategies (Mahler et al., 2014). If the home was to be recognized as a health-promoting setting, it would be pivotal to also focus on their abilities, safety, resources, and self-efficacy (Mahler et al., 2014). In summary, that review called for more-detailed knowledge of which aspects of living at home can improve health, since this is necessary to develop health-promoting strategies for older adults in that situation.

Regarding the meaning of home from a health perspective, Barry et al. (2018) emphasized the connection between attachment to home and how this affects the mental and physical health in concept analyses of women living at home alone. They found that breaking women’s attachment to home has been linked to loneliness, a yearning for home, and poor health.

Fänge and Ivanoff (2009) described an inner force that helps to maintain health in older people living at home, and that the home needed to be considered as the most-central place for health in the lives of old people. Hatcher et al. (2019) proposed a theory of “holding momentum” as a key strategy to maintaining the ability to live at home during old age. They emphasized that this momentum is largely dependent on an older person’s degree of resilience. The study’s implications for older people living at home centred on maintaining their health and independence. The authors suggested that focusing on health would support the ability of older people to continue living at home.

Beyond these early foundational studies, to our knowledge there has been little recent research into the health-promoting perspective of older people living at home. The desirability of living at home for as long as possible indicates a need to improve knowledge about how to enable older people to live a good life at home. Research is required to further elucidate the strategies that older people use to promote their health and allow them to live good lives at home. Narrative studies that centre on the voices of older individuals themselves are also scarce in the literature related to health promotion and home environments.

In Norway, as in many Western countries, there has been a significant investment in home care services to accommodate the growing older population ageing in place (OECD, 2023; 2023). 80% of the Norwegian population over 80 years are homeowners (Statistics Norway, 2023). Additionally, the demographic shift, particularly in rural areas, poses challenges related to workforce shortages. Ensuring adequate access to healthcare professionals in rural regions will be a key issue in the coming years (2023). To address this, there has been a deliberate focus on developing centrally located assistance care facilities and senior housing. This strategy aims to alleviate the workload of home care services by minimizing travel time between service users’ homes, and to increase the availability of safe housing options for the elderly in rural areas. However, many municipalities have yet to implement such initiatives (2023). Primary healthcare services in Norway are funded mainly by the government, and it is the responsibility of municipalities to provide them. Patients must apply for home care services, and a purchaser office determines service eligibility based on needs (Saunes, 2020). Regardless of age, financial situation, gender, social status, diagnosis, and family situation of applicants, the municipalities should provide reasonable, high-quality healthcare and social services to everyone who needs them. This includes various basic physical, mental, social, and spiritual needs (Health and Care Services Act, 2011). Home care nursing services in Norway are part of the public health system and are free of charge to the recipients while home care help like house maintenance incurs a means tested fee. Formal support refers to the help given by professionals within their mandate, such as home care services. Informal support refers to the help given by families, social networks, volunteers, and the community, which can include help with transportation, maintaining the house and garden, shovelling snow, socializing, shopping, and assistance with digital and technology challenges.

## Aim

Increasing longevity is resulting in people having to manage more complex health needs as they age. This makes it crucial to gain insights from older people themselves who already receive home care nursing and are experiencing health challenges. Thus, this study aimed to characterize the meaning of home from a health-promoting perspective for older people who are living at home and receiving home care services.

## A salutogenic orientation: the theoretical foundation of the study

To shed light on the health promotive dimensions of the home and of living at home, a salutogenic theoretical framework was found useful as a foundation for these narratives. Salutogenesis focuses on people’s abilities and is a resource-oriented approach (Haugan & Eriksson, 2021). According to Antonovsky’s salutogenic theory, health can be placed on a continuum between ease and “dis-ease”: all people are constantly moving within this continuum, and the aim is to move towards the “ease” end of it. Antonovsky’s theory was based on daily life constantly changing, with people needing to be able to understand their situation and use the resources available to them to optimize their situation. This ability is called the sense of coherence (SOC), which reflects an individual’s perception of life and their ability to cope with stressful situations (Antonovsky, 1987). This helps to explain why some people can remain healthy even in stressful situations. The SOC is a personal mindset that influences one’s thoughts, actions, and beliefs, creates a sense of inner trust, and consists of three aspects: comprehensibility, meaningfulness, and manageability. A strong SOC will allow the individual to manage stress, identify and mobilize their internal and external resources, find solutions, and resolve tensions in a healthier manner (Antonovsky, 1987). Antonovsky (1987)assumed that the SOC continued to develop up to the age of 30 years, remained stable until retirement, and then decreased thereafter. However, subsequent research has shown that the SOC develops throughout the lifespan, actually increasing monotonically with age (Koelen & Eriksson, 2021; Tan et al., 2014).

Generalized resistance resources (GRR) and specific resistance resources (SRR) are other key concepts in salutogenic theory. These resistance resources exist at different levels and represent the prerequisites for developing a strong SOC (Koelen & Eriksson, 2021). GRR are found within individuals as resources associated with their personal traits and abilities, but also within their immediate and distant environment, and can be both material and nonmaterial. On the other hand, SRR are resources that are useful in specific situations. It is not enough for resources to be available—individuals must be able to recognize, utilize, and reuse them to improve their health and well-being (Koelen & Eriksson, 2021).

## Materials and methods

### Study design

This study took a narrative approach to obtain older persons’ stories and reveal a nuanced and rich understanding of the meaning of home as a health-promoting setting (Riessman, 2008). Narrative gerontology involves researchers inviting older adults to share stories from their lives, often with the aid of research interviews. A fundamental assumption in this field is that identity development and meaning-making are ongoing processes that continue throughout the lifespan (Blix, 2016).

Storytelling is a powerful tool that allows the audience to connect with the narrator’s experiences and understand their perspective. Becker (1997) defined narratives “as the stories people tell about themselves.” These stories reflect their experiences and how they wish others to perceive them. A story is not neutral, instead being grounded in a specific cultural setting, interaction, and history. The central insight of a narrative study is that humans are fundamentally storytelling creatures—they are biographical beings as much as biological beings (Kenyon & Randall, 1999).

### Setting

This study was part of a larger research project (RCN number 320622) that has the overall aim of improving the knowledge of health determinants of older recipients of home care. One goal of this programme is to advance health-promoting practices in municipal healthcare services for older recipients of home care nursing. The present study focused on exploring the perspectives of older people themselves.

### Recruitment and participants

The following inclusion criteria were applied when recruiting the study participants: older than 75 years, receiving home care nursing for more than 1 year, and able to participate in an interview that would last for about 1 hour. The participants were selected purposefully to reflect a variety of characteristics in order to gain insight into different situations and characteristics. We aimed for the included participants to exhibit diversity in terms of marital status (married, single, or widowed), gender, living arrangement (alone or cohabitating), having, or not having children, and type of residence (detached house or apartment). All participants were recruited from two municipalities in South-Eastern Norway: one rural and one semiurban.

Participants were recruited by nurses working in the municipal home care services. Potential participants were approached and given a comprehensive oral briefing about the study as well as a folder containing written information about the study and then asked if they wished to participate. The first author was provided with the contact details of the participants who had confirmed their willingness to participate, and scheduled appointments with them. All participated with written informed consent.

The sample comprised seven women and three men aged between 79 and 103 years (Table 1).

**Table 1.** Characteristics of the sample.

Participant’s pseudonym	Age category, years	Gender	Cohabitating or living alone	Housing type	Informal caregivers	Home care visits per day
Astrid	75–80	Female	Alone	Apartment	Family	Five or more
Arne	80–90	Male	Alone	Detached house	Family	One
Anne	90+	Female	Alone	Apartment	Family	Four
Solveig	90+	Female	Cohabitating	Detached house	Spouse Family	One
Gerd	90+	Female	Alone	Detached house		Two
Else	80–90	Female	Alone	Detached house	Family	Three
Ragnhild	80–90	Female	Cohabitating	Detached house	Family	Two
Per	90+	Male	Cohabitating	Detached house	Family	One
Kjell	75–80	Male	Alone	Detached house		Three
Kari	90+	Female	Alone	Detached house	Family	Three

### The interviews

Ten individual narrative face-to-face interviews were conducted by the first author between February and April 2022. The interviews took place in the participants’ homes, which were in line with their wishes and also provided a place context. Seeing where the participants lived, hearing their views of home, and placing their narrative within the context of their home facilitated the ability to interpret their stories (de Medeiros & Rubinstein, 2015).

The interview guide was based on a thorough review of the literature. It focused on the following themes: participants’ experience and reflections of the meaning of home from multiple perspectives: personal, physical and emotional, and in relation to their health and age. The first draft of the guide was then discussed by the authors and two nurses from the home care nursing service. Thereafter a pilot interview was conducted with an older person receiving home care nursing. This led to minor modifications to the guide to make some questions more specific. The final interview guide served as an aide-mémoire, included questions such as: “When you think about your home today and what it means to you, what are your immediate thoughts? Can you describe what is important to you in your home? What does your home mean to you after your health has worsened? What does your home mean to you as you have grown older?” The continuous analysis confirmed the relevance of the themes. Moreover, the quality of the interviews was enhanced by encouraging the participants to introduce themes on their own to convey all relevant aspects of their experiences. The interviews lasted from 40 to 120 minutes, were audio recorded and transcribed verbatim by the first and fourth authors. Due to the time of the recruitment process and the continuously analysing process the sample size was consecutively considered. Given the depth and quality of the interview data, along with purposeful sampling, we concluded that meaning saturation was reached after conducting ten interviews (Ahmed, 2025).

### Narrative thematic analysis

The analysis primarily employed a thematic approach focusing on identifying patterns and key themes across the dataset. However, care was taken to preserve the integrity of each participant’s account, ensuring that their narratives were understood within the context of their broader life stories and situated experiences.

The stories were centred around the context of home, and how it was to live and age at home. The unit of analysis was what they said about their everyday life at home as described by Riessman (2008). We chose to maintain the episodic form of the narrative in presenting our result, ensuring coherence along thematic lines rather than following a dramaturgical structure (Hyvärinen et al., 2010). We have tried to keep the interview segments rich in order to improve the context and trustworthiness. In narrative research saturation is achieved when the data collected provides a thorough and coherent understanding of the participants’ stories and experiences (Ahmed, 2025). This approach enabled us to achieve saturation in our data collection.

The analysis started immediately after the interviews had been conducted so that the impressions from the interviews could be recorded accurately. Data files were created, organized, and downloaded to NVivo software. The texts were read through, and margin notes and initial codes were added. In the coding process we were data led with an inductive approach, but as researchers we brought with us different perspectives. Thus, the analysis had elements of both deductive and inductive orientations (Braun & Clarke, 2022).

The first author had the main responsibility for conducting the analysis, with all authors participating in all stages of the analysis process. Both first and last authors read all the interviews and discussed the findings. The second, third, and fourth authors read a subset of the interviews. In the development from codes to themes we were looking for patterns and similarities related to our research question both in each interview and across the interviews. Our iterative process involved moving back and forth between the whole and parts of the data (Kvale & Brinkmann, 2009). Based on this, the first author subsequently developed an initial draft of themes. All authors then engaged in discussions to refine these into primary theme and subthemes. The interviews were important to understand the context of the participants and a separate summary for each participant was written.

To provide trustworthiness and to ensure a comprehensive and nuanced understanding of each theme, we carefully selected excerpts from the narratives to illustrate the findings and provide diversity. All participants made valuable contributions to the results.

### Ethical considerations

The Regional Committees for Medical and Health Research Ethics in Norway considered that the study fell outside their remit and did not require approval (reference number 398854). The Norwegian Agency for Shared Services in Education and Research approved the study (reference number 689856). To ensure the privacy and confidentiality of the participants, all identifiable information such as places, names, and dates was anonymized, and pseudonyms were assigned to the participants. Additional oral information was provided during each interview to reassure the participant of their anonymity and their right to withdraw from the study at any point without any impact on the future support that they would receive from the home care services. Written consents were obtained from the participants before the interviews.

## Results

**Figure 1.**
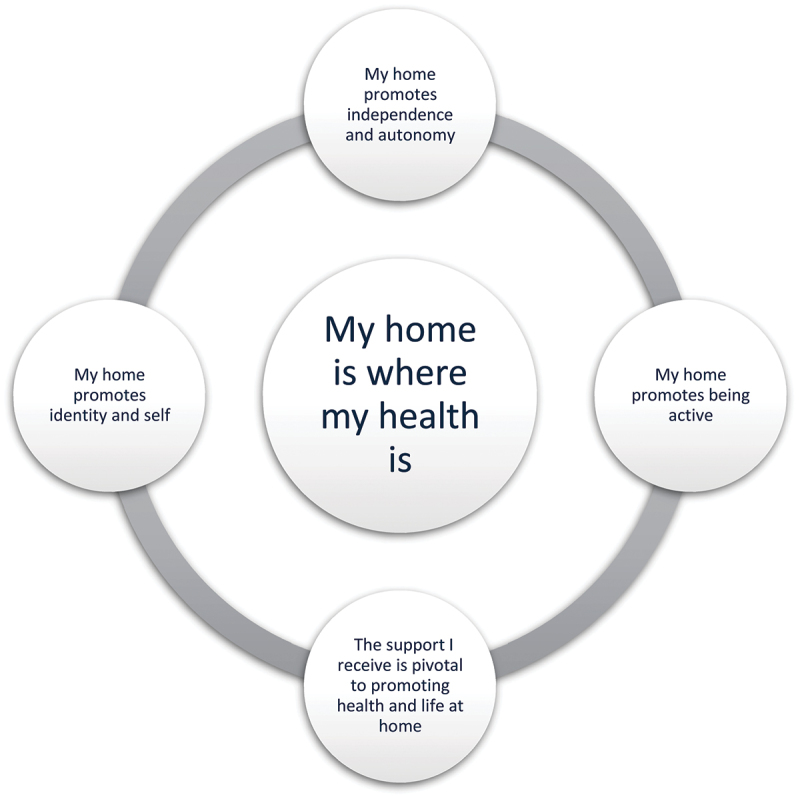
Overview of the primary theme and the subthemes.

The primary theme identified was “My home is where my health is.” It reflects a compellation of the four subthemes emerging from the data: (1) my home promotes independence and autonomy, (2) my home promotes identity and self, (3) my home promotes being active, and (4) the support I receive is pivotal to promoting health and life at home. See Figure 1. It should be emphasized that home refers to where the participants are living, and it was important to include participants covering a variety of common housing for older persons (houses and flats, original homes and new homes). Further, from the participants narratives, they elaborated that the term home was where they lived, but that it also included their immediate surroundings like their gardens and sometimes also their neighbourhood.

### My home promotes independence and autonomy

The participants placed great importance on maintaining their independence and autonomy. Living at home was a key factor in achieving this, with it promoting confidence in their ability to take care of themselves. They talked about the importance of being the one in control of the decisions in their own lives. This included minor decisions such when to get up in the morning, what to have for dinner, and when to entertain guests.

Astrid lived in a third-floor apartment. She was a stroke survivor and needed extended help from the home care services and was dependent on using a wheelchair. After the stroke she had a longer stay at a nursing home for rehabilitation. Although she had positive experiences during her institutional stay, it was not where she felt that she belonged. Due to challenges with stairs in her old home her children had helped her move into an apartment suitable for wheelchair users. She appreciated being able to live independently at home and being in charge of her own decisions and routines. Astrid explained:

You know what? I feel more whole by living in my own home. This is where I belong even if there are things that could have been better. I belong here. … It’s so lovely that I can choose. I can choose if I want to go to the toilet or not. Or when I want to eat or not. I can’t do that at the nursing home.

Like Astrid, Arne lived alone, but in a detached house in a semiurban neighbourhood. He had problems with his vision and was unsteady. He had a walker but preferred not to use it. He considered that he had worked hard on obtaining his home in his younger years:

It is very important to stay at home … Yes, I have my own house and that’s important to me. Therefore, everybody who wants to stay at home must be allowed to and I think when you receive home care now and then you can do it … . Everybody should be allowed to live in their own nest. (Arne)

Despite the physical declines associated with ageing and recent deteriorations in health, the sense of being independent and autonomous was strong among the participants living at home. This sense of independence and autonomy contributed to their motivation and self-confidence. However, the participants’ independence and autonomy were challenged in different ways.

Astrid considered staying at home, but the children insisted she needed the care provided at the nursing home due to her health and physical limitations. Nevertheless, she wanted to discover how she would manage on her own. Her inner motivation for staying home was strong, as was her belief that she could make it herself with some help from the home care services and her children. She knew that things would not be the same as before, but she had a belief that the outcome would be good. After the decision was made, her children continued to discuss it:

My children say that at the nursing home, you can have everything. No, I will not, I say. I cannot furnish my room as I like. I think the boys would think it was very nice if I stayed at the nursing home, since then they didn’t have to buy the groceries for me and all that. They said I was a naggy mother, but as long as they have me, they’ll have to live with it. It is important to me that I have a small apartment. If you had asked me when I stayed at the nursing home, I would have said that it isn’t where I want to live even if that’s what my boys want me to do. (Astrid)

Many participants reported feeling challenged by being dependent on help from their family and at the same time had different judgements about whether they could manage to live at home alone. Differences in opinions with their caregivers added to the feeling of being a burden when asking for help, which resulted in a form of imbalance in their relationships with their families. Moreover, standing up for oneself also showed strength and manifested will to stay at home and decide for themselves.

The participants expressed a strong desire to maintain their independence and autonomy while living at home. They regarded their homes as crucial to achieving this objective. Being able to live at home and make everyday decisions about daily life without interference was highly valued.

### My home promotes identity and self

Living at home was an important part of maintaining the identity and self for the participants. They had experienced numerous losses, including health, family, friends and opportunities to engage in activities they once enjoyed. Being in their own home reminded them of who they had been and who they still were. This sense of belonging- to move in familiar spaces and be surrounded by familiar, cherished things enabled them to maintain their identity while alone also strengthened the self when they were experiencing life challenges.

Being among their belongings that had value to them in a place where memories of a past life were present was important to several of the participants. Some of them had lived in their home for 40–60 years, which resulted in a particularly strong attachment to the home. The home was significant for the memories where they had lived with their family, got married, and raised their children. They wanted to continue living at home, where they had been for such a long time. They felt safe in the familiar surroundings that they knew so well and expressed that this promoted a sense of security, comfort, and meaningfulness.

Ragnhild lived with her husband in a large house in a rural area. Ragnhild considered it important to live together with her husband, and she expressed a strong attachment to their home where they had lived since they got married. She had recently been discharged from a hospital after a long stay due to acute illness and was pleased to be home again. She needed a considerable amount of help from her husband and the home care services. The hospital stay had set her back, but her physical mobility had improved during several weeks after returning to her home and she was hopeful about the future. Ragnhild narrated:

Yes, here it is so good, here I want to stay. Nobody can say that I must move. No, I’m fine. I have lived here for 50 years, imagine that. We built the house here and the kids have grown up here. So, it’s—that’s all I can say about that. Yes, all my clutter I have here.

The participants described how they spent time remembering events in their lives, people they had loved, and books they had read. Thinking about these memories gave pleasure to many of them, and this was closely connected to their physical home and its surroundings. The home represented a comfortable, secure, and safe space, a place where they were connected to both their present and past selves.

A notable aspect in the narratives of home was understanding that their home was changeable: for some it was the home they had lived in for years, while for others it could be a new apartment, but it had to be *their* home and not an institution. Memories connected to the home could be transferred when moving to a new home. Astrid had lived in her apartment for about 2 years and described how she had brought with her things from her former apartment that were important to her. Things that she had saved up for during her life and things that she had inherited now gave the new home a deeper value:

Yes, among other things what my dad has carved and what he has painted and books, I love that. I don’t have many things here, but I can make the home care nurses get things for me if I want to read something. And then I can see; there is my dad’s painted stuff and some pictures and stuff, yes that’s wonderful. (Astrid)

The home was also important for the identity. This seemed to be particularly important when they had experienced reduced roles and impairments of physical abilities and health. Being among familiar things and feeling attached to their home was an important aspect of promoting identity and self.

### My home promotes being active

The participants considered their home to be an important place to maintain a meaningful everyday life and to perform their daily routines and do what was necessary to be able to keep their home. This daily activity was important for their well-being and for promoting their health. Living at home allowed them to be active in different ways that fit their personality, physical functioning, and home environment. Being in the privacy of their home allowed them to do as little or as much as they wanted to do when they wanted to. These everyday activities comprised physical, psychological and cognitive activities, like outdoor activities such as chopping wood, mowing grass, and walking, and indoor activities such as cooking, cleaning, vacuuming, listening to the radio, reading a book. The activities essentially formed an integrated part of the home and of living there. Their knowledge of the surroundings meant that they could plan and do activities based on their experience and how they were feeling on the day. Living at home also gave them motivation to do things for themselves.

The everyday activities had value to many of the participants beyond the activities themselves as a cherished part of life, including the routine giving them life meaning and continuity and making things manageable. Taking care of home was also taking care of oneself. Thinking about the activities was an important part of the experience, and planning what to do and when to do it was meaningful to the participants. This also seemed to serve as a connection to their past as it was something that they could keep on doing.

Gerd had lived alone for more than 30 years. She was a widow and lived in a house with three floors and a garden in a semiurban neighbourhood. She did not have children, but she had a brother she was close to. She occasionally used a walker, mostly when she was outdoors. Gerd kept herself busy with daily activities and talked about the importance of not sitting still. She cleaned the house herself, baked bread, gardened, and liked to read books. Taking care of her house was important to her from a health-promoting perspective because it kept her active and moving and so helped her to remain in a good condition both physically and psychologically. Gerd narrated:

Yes, that I shall do everything myself. That’s me. Of course, I’m sure it’s nice to get the food delivered at the door, but then you don’t get to move. That’s everything, you must move … . I grew up on a farm so that’s why I’m doing so well, because I’m used to working, and I cannot just sit down and do nothing. I have a big garden as well, but I don’t know if I can cope with it. I cannot refrain from tending to it you know. So, as I say if you have a house there is always something to do—always.

On the other hand, Gerd said that she also had to consider what she could not do anymore. This was also the case with the other participants, who described a daily life where diminishing ability, more pain and health problems, and becoming frailer forced them to find strategies to compensate for their impairments. This was a continuous and ongoing challenge that they had to address every day.

Anne was also a widow living in a spacious apartment in the town centre. It was furnished with items from her former home, and she had most of her family close by. She was in fairly good physical condition, but she had broken her arm during the previous year, which had been painful and had reduced her physical abilities. She also had the limitation of reduced vision. Anne explained as follows:

Because of my vision I can’t read magazines anymore. … Now I have problems, I don’t keep the newspaper anymore, I read the obituary. And the headings, but now I don’t want to keep it anymore. The TV is enough. I can hear, but I can’t see clearly you know. That’s not agreeable, no, in the evening I get very … the tears flow. Heck no. I can’t do what I did before. No, I don’t. I do just what is … I clean the floor, I vacuum what I can, but then many times … I have some pain in my arm so I can’t … many times I just give up when I am doing something, so I do little by little. … if it’s not ok here, please don’t look at the dust. I’m dusting when I can bear it and want to.

Their diminished physical ability was a part of the lives of the participants that they could not escape from. They described how they had to think, plan, and adapt to new solutions for how to do things. Sometimes they had to accept that they could not get everything done like they used to. The participants described that they had to balance the need to do something with what they could manage to do. They wanted to do what they always had done, but physical limitations would often prevent them from doing things, and they had to consider the consequences related to their abilities, energy levels, and the possibility of hurting themselves. Some of them described how they sometimes took risks even when they knew what the consequences might be. Two of the women talked about broken arms and ribs caused by cleaning windows and roofs, and how they now had to think twice when performing certain physical activities. Gerd narrated:

Yes, it is a bit hurtful for me. The motivation is there, but then it will go wrong. So it is, the body is old of course, it can’t endure everything. The thing is, I don’t feel that old, luckily, but then everything is old, that’s the weird thing…My body is so fragile and so little needs to go wrong, but I must endure it. I should have been out for a walk today, but it has been so slippery. That’s dangerous.

As Gerd mentioned, the different seasons influenced which activities could be performed, with ice and snow during the winter being an obstacle to getting out of the house for many of the participants. They tended to be less active during winter and looked forward to spring and summer when they could go out of the house again. The snow and ice on the roads and sidewalks during wintertime resulted in several of the participants not going out if they could avoid it due to the risk of falling on the ice. The participants tailored their approaches to compensate for their losses and impairments, which included accepting their situation, balancing their abilities and limitations, and adapting their activities as part of their daily lives. This included, for example, reducing activities, acquiring assistive devices, avoiding outdoor walks during winter, preparing simpler meals or distributing tasks throughout the day. Living at home in a familiar environment enabled them to remain active in various ways, engaging in a range of physical, psychological and cognitive activities. These aspects contributed to their well-being and health.

### The support I receive is pivotal to promoting health and life at home

To make it possible to live at home, the participants needed support to compensate for their physical impairments. This was important for being able to maintain an adequate life and well-being. The participants’ narratives about deteriorating health and a failing body combined with living at home and taking care of themselves constituted descriptions of continuous work.

Per lived in a rural area with his wife who was suffering from dementia. They had a large house that they had fitted with devices for supporting the ageing process, such as having everything they needed on one floor. Per had problems with his legs and was dependent on a walker. Because of his wife’s illness, he had taken responsibility for everything. This was challenging for Per since the house had previously been his wife’s domain. They needed practical help in the house and had regular and fixed agreements with their daughter to provide support. Per explained:

And then we have a great daughter who helps us and washes our clothes. She is our private secretary and confidential supervisor. She fixes everything for us. You are dependent on people to pick up and bring things. Yes, and she orders our groceries online which are then delivered to the door. We usually get three bags with groceries that we must put away. I have my walker so I can take the bags on the walker one by one. That works, I think it works quite all right actually. It was easier before I got so impaired of course. But that’s how it is.

The participants expressed their gratitude and satisfaction about the help provided by the home care services, but these could not cover all of their needs. For example, the home care services provided help with buying groceries, but some of the participants also needed help with other purchases, such as when they needed to buy a new vacuum cleaner.

The support required varied markedly depending on the health challenges and physical disabilities of individual participants, but also with their housing situation. Having what they needed on one floor made it easier for them to cope by themselves, while something like a narrow door could make them dependent on help to do the laundry and even to get out of the front door.

Participants such as Per who had fixed agreements and knew that things would be done were very content with the situation. It made them feel safe and gave them confidence that things were planned and would be done. Fixed agreements for informal support also seemed to contribute to the safety and satisfaction that things would be taken care of.

Kari lived alone in a large house with a garden in a rural neighbourhood. She was a widow and had lived alone for more than 20 years. She was an energetic woman who liked to cook and bake and to keep her house tidy. She had a walker but rarely used it. She could no longer go for longer walks outside due to problems with her legs, but she reported that she experienced no problems moving around in the house or climbing stairs. Kari did not have any fixed agreement for informal support, and she worried about how to get the help she needed. She wanted to do the grocery shopping herself because finding good bargains and selecting specific products was important to her. However, she needed help with transportation, and that was outside the remit of home care services. She used to get help from her son, but he could no longer provide this due to his own health problems. Not knowing how to get things done contributed to stress and insecurity, and Kari expressed that she felt like a burden and like she was nagging about it to her family:

Yes, because my son says that I can just order the grocery on the phone. He used to help me with the shopping, but he could not keep on doing that. So now I got the neighbour to drive me down to the store on Friday and I did a good deal of grocery shopping and then I don’t know how it will be next week. So, no, I could just order the grocery, but he said he could not help me with it. (Kari)

Most of the participants had to rely on their families and sometimes neighbours and friends for the informal support that they needed. Some of the participants would pay for private services, but not all of them could afford that.

Astrid wanted more help from her children than they gave her. She said that she had given her car to one of her sons after she had stopped driving. She had also given her children money after a real estate sale. She had hoped that giving these gifts would lead to more help in exchange and expressed her disappointment that this was not forthcoming. Differing expectations between participants and families about support caused difficulties. It is important to note that some of the children mentioned in these narratives were approaching retirement themselves or had their own health issues, and so did not have the ability to help as much as was needed.

The participants shared their reflections and worries about how to get help with tasks they could no longer perform for themselves. All of the participants were grateful for the help they got from both their formal and informal caregivers and supporters. They emphasized that this support was fundamental to being able to live at home in old age with health challenges.

## Discussion

This study explored the meaning of home from a health-promoting perspective for older adults living at home. The primary theme in this study was that the home is where the health is. The main finding was that living at home enhances feelings of autonomy and independence, holds attachment and memories, and motivates activity, all of which seem pivotal to health promotion and well-being in old age. The connection between home and health is not new, with several studies having already suggested this in different ways (Koelen & Lindström, 2005; Kylén et al., 2019; Rowles & Bernard, 2013). This study has described how older persons themselves perceive the home as a place that contributes to promoting their health, through the maintenance of ordinary, trivial activities -both physical, psychological and cognitive. We consider this knowledge to be significant for healthcare professionals responsible for older people living at home, for example, by paying attention to and recognizing their resources and capacities to plan and keep the home in order. The present study’s contribution is a further expansion of knowledge in detailing the significance of the home as central in a health promotive perspective. The study identified health promotive characteristics of the home and living at home for the participants. How living at home is health promotive will be discussed in the section below.

### It is best to live at home, where I can do as I please and decide things for myself

One of the main findings was that their home was a place where the participants were autonomous and independent. This was very important to all of the participants. The emphasis on autonomy and independence is consistent with findings from other studies involving older people living at home (Hatcher et al., 2019; Herrler et al., 2022; Wiles et al., 2012). However, the present results also indicate that the feeling of autonomy and independence were flexible concepts. Many of the participants had severe health challenges and were dependent on support from both formal and informal caregivers. Despite this, they still expressed feelings of autonomy and independence, and they still found it worthwhile to live in their own homes. In addition, four of the participants had experienced living in an institution for a short time and compared their present life at home with life at an institution where they had to adapt to routines decided by others and in a new environment. They emphasized the contrast between their life now, where they could decide everything themselves, and that in an institution where they lived a more-passive life and had much less autonomy. Furthermore, some of the participants mentioned that they also needed their autonomy and strength when they sometimes had to stand up for themselves in discussions with their families. Several of the participants had had discussions with their families about housing, where the family emphasized safety and the participants emphasized autonomy; that is, the older person wanted to live with autonomy at home whereas the family wanted to apply for an institution or sheltered housing. While participants acknowledged the presence of safety concerns, they expressed a strong desire to maintain their independence by continuing to live in their own homes. Such differences between older persons and their family caregivers are seldom addressed from the perspective of the older persons. However, literature on the perspectives of family caregivers has emphasized the ambivalence and conflict between autonomy versus taking over responsibility of the older person (Luichies et al., 2021). Most participants in the present study talked about this discreetly and indirectly, which suggests that it was a sensitive and difficult subject to address. Their dependency could make it difficult to advocate their opinions because they knew their decisions would have an impact on their caregivers. Recognizing oneself as an individual capable of making choices and arrangements based on subjective preferences, independent of external constraints and limitations, holds significance at any stage of life (Vasara, 2015).

Living at home allowed our participants to perform meaningful activities, which was particularly significant for them. Performing daily activities promoted health by contributing to both physical activity and a meaningful life. It was particularly interesting that the ability to plan activities for today, tomorrow, and next month was also highly important to the participants. Thinking about and planning activities was an important part of this, which also made the activities more complex. The emphasis on activity corresponds with previous findings (Dahlin-Ivanoff et al., 2007; Fänge & Ivanoff, 2009; Kylén et al., 2019; Seah et al., 2021; J. Sixsmith et al., 2014; Søvde et al., 2022). Bigonnesse et al. (2014) found that the activities of daily living played important roles in the everyday lives and the meaning of home in older adults. Kylén et al. (2019) found that activities performed at home were seen as self-rewarding and seemed to contribute to well-being. However, the emphasis on planning and thinking about the activities is a novel finding of the present study. The planning itself was an important part of the activity. The ability to anticipate and plan reflects an optimistic outlook on life and is an important aspect of living at home. Good health has previously been linked to acceptance and viewing adjustments with optimism among older adults (Bryant et al., 2001; Song & Kong, 2015).

Considering the theoretical underpinnings of the present study, autonomy, independence, and the motivation to be active are resources that helped the participants to deal with challenges in their lives and may contribute to developing a stronger SOC (Antonovsky, 1987). Being active allowed the participants to control their routines and gave them a sense of continuity with the past in a life that was constantly changing because of old age and deteriorating health.

We observed in the narratives that activities also allowed the participants to focus on positive aspects and what worked for them even if they were experiencing health challenges. The participants’ stories were similar to the findings of Fänge and Ivanoff (2009), who described that their participants challenged themselves in different ways and were persuaded that this was beneficial for their health.

A common feature of the participants’ stories was that they all had to stop performing certain activities due to their reduced physical ability and deteriorating health. However, they had adapted to the changes in their lives and accepted them as a natural part of ageing. They employed different strategies to compensate for their impairments, but they did all try to do something. The narratives about activity, planning, and the adaptations that the participants constantly applied in their lives correspond with the description of “holding momentum” as a process of continual decision-making, re-evaluating, and making new decisions about ageing at home—a process of working hard to stay in the same place (Hatcher et al., 2019). Having a feeling of autonomy and independence and being in control of one’s resources strengthened the feeling among the participants that they could manage their own lives. They all were remarkably focused on what worked rather than dwelling on what did not work. Attempting to remain at home provided a purpose and enhanced their SOC (Tan et al., 2014).

### I feel happy in my home

This study has also revealed the importance of memories connected to the home. Thinking about the past, the people they had cared for, the things they had achieved, and the activities they had done were important to them and seemed to contribute to their well-being. Home was their place where they could manage themselves, be private, and be themselves, which is consistent with previous findings (Aliakbarzadeh Arani et al., 2022; Coleman & Wiles, 2020; D. G. Rowles & Chaudrey, 2005). It is also in line with Romaniuk and Romaniuk (1981) reporting that recalling pleasant memories helped to pass the time of the day and could be amusing, entertaining, and informative, because it highlighted the successes and accomplishments of one’s life. As reported by Antonovsky (1987), the meaningfulness in their life was enhanced by memories connected to the home and home also strengthening their identity and self.

The participants expressed an attachment to their homes, surroundings, and cherished objects. The attachment was an emotional and positive bond between the participants and their homes, as also reported previously (Aliakbarzadeh Arani et al., 2022; Coleman & Wiles, 2020; Kylén et al., 2019; Rollero & De Piccoli, 2010; Swenson, 1998). Cherished items play a significant role in preserving personal and generational continuity (Tobin, 1996). These objects can bring back memories of deceased loved ones, and their value lies in their ability to create a SOC and connectedness across multiple generations. Therefore, they serve as markers of continuity and shared experiences (Tobin, 1996).

Continuity theory in gerontology posits that older individuals tend to seek continuity (Atchley, 1989). An experience of continuity helps people to reinforce their established selves, allowing them to perceive themselves as still aligned with their core identity, even amidst significant changes in their lives (Atchley, 1989; Thorsen, 1998). This is consistent with memories of and attachment to their home helping the present participants to integrate their past experiences into their present self and that reflects on positive memories and achievements contributed to their well-being. This is consistent with the research performed by Rowles and Bernard (2013). Sixsmith and Sixsmith (1991) claimed that having a sense of attachment to home is positively related to physical, social, and emotional health and well-being in older people. J. Sixsmith (1986) reported that the home serves as a central emotional and physical reference point in one’s life that reflects desires, feelings, hopes, and actions. The importance of this attachment as people age cannot be overstated, especially considering the physical limitations associated with ageing (D. G. Rowles & Chaudrey, 2005). However, our study showed that attachment to place was not solely connected to the original home but could also be achieved in a seniors’ apartment. This was also demonstrated by Munkejord et al. (2018), whose participants even reflected on why they had not taken this decision before because they now felt safer after moving to a new place, and some had even experienced health improvements. Leith (2006) found that place attachment when relocating to new accommodation was associated with whether the decision to relocate was taken autonomously and independently. When the participants were able to approve both the choice of location and the decision itself, they formed an initial attachment to their new home that counterbalanced their ambiguous feelings about the move. This is important since many older persons will have to move to a facility with a higher level of care and hence reduced autonomy. The present findings indicate that this can be done in a manner that addresses an older persons’ autonomy and attachment to home. This is essential knowledge to consider in order to enhance older individuals’ well-being and sense of belonging when they choose to relocate to other living facilities such as assisted living or nursing homes.

The strengthening of continuity, identity, and self by memories of and the attachment to home and cherished objects that was apparent in the stories of our participants was a prerequisite for developing a strong SOC and had a positive impact on the health and well-being of the participants. The home provided a basis for consistency and resources as older people confronted the uncertainties that came with old age (Haak et al., 2011). However, other studies have demonstrated that not everyone wants to remain in their original home, which could be due to various factors such as the condition of the home, declining health, and not feeling safe anymore (Munkejord et al., 2018).

### However, I cannot do it without help and support

One important finding of our study was the influence of both formal and informal support. The participants made it clear that they were dependent on support to be able to live at home. Furthermore, access to informal support was particularly challenging for those who did not have family or others nearby who could help them. The children of some of the oldest participants were either approaching retirement themselves or had already retired and some had their own health problems that made it difficult to provide the amount of support that was needed.

Some of the participants in our study were not able to leave the house independently. This particularly affected the ability to have a social life, which Tan et al. (2014) reported might have a huge impact on health and well-being for older persons given the importance of support and social relationships.

The participants who had challenges with getting this support did spend time, attention, and energy on trying to solve the problem, including who to ask and what to do. Access to informal support has previously been reported to be a problem (Bigonnesse et al., 2014; Bjerkmo et al., 2021; Munkejord et al., 2018). Borglin et al. (2006) found that older persons with fewer opportunities to receive a “helping hand” were likely to have a lower SOC. Munkejord et al. (2018) found that their participants who had moved from their original home to a seniors’ apartment or an assisted-living facility tended to be those that had received relatively little informal support. The amount of informal support received seemed to be particularly unstable for older people without family nearby. In Norway, this may become a challenge, especially in rural areas where ageing will be most pronounced and occur most rapidly, due to the trend where young people move to more central regions while the elderly remain (2018). With the consequences of less informal support being available. More people are living alone, and the number of older people without family providing for their informal care will probably increase in the future. Addressing the anticipated need for informal support is crucial in the planning of home care services, especially considering certain caregiver groups -particularly those managing additional responsibilities alongside caregiving, experience negative health effects of caregiving (Bom et al., 2019).

Knowledge about, access to, and the ability to use resources such as support and practical help are important components of the resistance resources needed to combat stressors in old age (Antonovsky, 1987). When older people do not have resistance resources, they might experience life as unpredictable and unmanageable. Antonovsky viewed this as not having enough resistance resources, resulting in older people not perceiving life as manageable, meaningful, and comprehensible. This can lead to them worrying about how to solve their challenges over both the short and long term (Espnes et al., 2021).

Our study has revealed that it is crucial to provide older adults living at home with health promotive services that equip them with the necessary resources to move towards the ease end of the ease and dis-ease continuum (Antonovsky, 1987). Furthermore, ageing in place should not become an excuse for underdeveloped health services for older individuals residing at home or an excuse for not developing institutional care facilities, as the participants in this study revealed that for many living at home it is hard work that demands significant resources. It is also important to note their significant dependence on the support provided by home care nursing. In addition, future strategies for developing services should recognize the positive aspects of living in one’s own home and focus on the resources of older adults rather than solely on their deficiencies. Older adults have a strong desire and motivation to remain at home, and home care nurses should support this aspiration. For example, interventions aimed at home-dwelling older people should be implemented earlier to provide necessary support before extensive assistance is required. This proactive approach may help prevent situations where more comprehensive care becomes necessary. This study also highlights the need for cross-sectoral collaboration and support, extending beyond the healthcare sector to include enhanced provisions for transportation and facility-related services such as cleaning and maintenance. Additionally, to enhance the health and well-being of older adults living at home and to prevent passive responses to emerging challenges, it is crucial to strengthen health promotion within home care nursing. By empowering individuals, home care nurses can support older adults in leading health promotive lives, thereby fostering independence and enabling them to utilize their own resources.

This study adds new to the knowledge about the home as a place for health promotion by exploring the health promotive dimensions of the home and everyday life. By focusing on older individuals who face health challenges and receive home nursing, we enhance our knowledge in this area. Additionally, capturing the perspectives of older adults themselves was valuable, especially given the ongoing development of home care services.

## Strengths and limitations

One important strength of the present study was the rich material provided by the narrative interviews. The participants generously shared their opinions in the narrative interviews, which provided a broad insight into their lives and their perspectives on living at home and being dependent on help. The cooperation with the home care services was also another strength of this study since it allowed the inclusion of older people who lived relatively isolated lives at home and would not have been possible to achieve otherwise. This gave valuable insight into the lives of older people who did not participate much in society or in social activities outside of their homes any longer.

This study was also subject to some limitations. First, the sample of 10 participants may limit the transferability to other populations, like older persons with immigrant backgrounds. Second, the participants expressed positive attitudes towards living at home; however, the results might differ for a sample with more diverse opinions on the matter. While the home is often idealized as a sanctuary of safety and support, this image may not align with the lived experiences of all individuals. Third, the open questioning in the narrative interviews might have suppressed other nuances, which can be considered a methodological limitation. Fourth, the participants were all homeowners, which may have impacted on their opinions about living at home.

## Conclusion

We interviewed older adults to explore the meaning of home from a health-promoting perspective and how health can be promoted by specific characteristics of everyday life. The narratives revealed that the meaning of home enhanced feelings of autonomy, independence, and attachment, as well as improving memories and motivating activity. They considered the home to be where their health is, which suggests that living at home is pivotal to health promotion and well-being in old age. From a salutogenic perspective, living at home with a positive outlook, and good support contributes to comprehensibility, manageability, and meaningfulness. This is in line with previous research and could contribute to a stronger SOC and thereby be a strategy for health promotion and well-being in old age. Future strategies for tailoring care services should consider that older individuals with complex health conditions require both formal and informal care and support, even when living at home. Furthermore, this study underscores the importance of enabling home-dwelling older adults to utilize their own capacities. In developing future home care services, it is crucial to focus on enhancing these capacities.

## Data Availability

The data are not publicly available due to privacy and ethical restrictions.
